# German Reviews

**Published:** 1891-09

**Authors:** Leonard Pearson


### Selections from Foreign Journals.

### *GERMAN.*

By Leonard Pearson, B. S., V. M. D.

### Treatment of Bog Spavins.

Prof. Hoffmann, Stuttgart.

*(Repert. der Thierheilk. 52, 4.)*

The most important point in the treatment of all blemishes of this class is rest; if necessary a sling. The hock should be made immobile by a bandage of plastic felt, plaster of Paris or strips of leather and a rubber bandage. In fresh cases pressure is of the greatest service and can be most conveniently applied by laying a bunch of jute or oakum, as large as the fist, on each side of the dilatation and covering them with a rubber bandage, run from below upward and tightly drawn. Such a bandage should not remain longer than 2—3 hours and after its removal the skin must be rubbed with a liniment composed of equal parts of alcohol, ammonia and turpentine. After 8 hours the bandage may be again applied, for two hours, and the liniment again used after its removal. The bandaging and rubbing may thus be alternated until a general swelling surrounds the joints, after which the bog-spavin often disappears. During this treatment it is advised to give the horse only dry food and water but once in three days. Blisters have also given good results, in fresh cases, but the water treatment and massage can not be recommended.

When the gall is hard and painful the above treatment may relieve the pain but does not remove the enlargement. Neither blisters, "absorbing ointments," the firing iron nor setons give the wished-for result. The treatment must directly affect the synovial membrane of the joint. The best method of applying this treatment is to place the horse in stocks, shave and disinfect a small part of the surface of the gall, lightly burn a spot the size of a pea (for the sake of more perfect disinfection), introduce a hypodermic needle and draw off the contents of the point with an aspirator. 10 gms. of a solution of iodoform in ether 1—10. is at once injected, through the same needle; the needle withdrawn and the small hole closed with a hot iron. The horse is now placed in slings, pads of jute soaked in carbolic acid and water applied to the joint and the rubber bandage put on, followed by the liniment, as described above. When this operation is antiseptically performed it is absolutely without danger. In 2—3 days the bog spavin always reappears, in its former dimentions, and the operation must be repeated for 4 to 6 weeks, at the end of which small galls will have disappeared and the large ones, if not cured, will be much reduced in size. If the wound becomes infected with septic organisms and suppurates the joint capsule must be freely opened, at its most dependent points and drained. The result of such an accident is invariably a stiff joint and the horse will be fit for nothing but slow work This complication may be prevented by rigid antiseptic precautions.

In contrast to the great danger of opening a joint, by incision, the tendinous sheaths may, with the assistance of antiseptics, be opened in this way with comparatively little risk. *Berl. Thierärz. Wochenschrift, No. 30, 1891.*

---

### A Case of Tuberculosis in a Horse.

Army Veterinarian Lebbin.

The reporter was called to see a horse with the following history: The animal had been losing flesh for six weeks; had a dull cough, especially when in the open air; showed difficulty of respiration and had had a few light attacks of colic. The bowels were very irregular; diarrhœa and constipation alternated. For the last 3 weeks the horse had refused his oats and ate nothing but a little clover hay.

The examination showed the patient to be a horse, 12 years old; hair dull and rough; flanks hollow; temperature 31.9° C.; pulse 58, weak and irregular; respiration 30 and oppressed and the conjunctiva pale. Percussion revealed several small dull spots in the lungs and auscultation bronchial, crepitant and friction sounds. Pressure upon the upper end of the trachea caused a weak, painful cough. Peristalsis was active.

In spite of the treatment and care the animal died the 13th day after the first visit. The autopsy was made six hours after death and showed the following: the lungs weighed one-half more than normal, and the lower portions felt firm. The cut surface was studded with small nodules, the size of a millet seed or pin head, of a yellowish-white color and the centre was calcified. The bronchial lymph glands were enlarged to the size of a man's fist, were of a firm consistency, the cross section was grey and sprinkled with small yellowish nodules. The bronchial mucus membrane showed catarrh. The costal pleura was covered here and there, with wart-like growths of a grey color, connected with one another by bands of connective tissue. The liver was enlarged and contained numerous nodules, the size of a pin head to that of a pea, with a calcified centre. The spleen showed the same lesions, but not to such an extent as the liver. The portal lymph glands were swollen and firm. The omentum contained nodules the size of a walnut, which were calcified at the centre. The mesenteric lymph glands were the size of the fist and showed the same changes as the bronchial glands. The peritoneum was healthy. As a result of these observations the diagnosis of Tuberculosis was made and was confirmed by the microscopic examinations. The tubercle bacilli were plentiful in the cheesy nodules of the lungs and in the nodes of the liver, spleen and affected lymphatic glands. Eighteen preparations were stained according to the Koch-Ehrlich method and B.-tuberculosis discovered in fourteen of them. *Zeitschrift für Veterinärkunde.* No. 2. iii.

---

### Wounds of the Tendons.

Army Veterinarian Goldmann.

A young cavalry horse was observed to have a wound of ½ in. diameter on the outside of the near hind cannon, near the fetlock and penetrating between the perforans and perforatus tendons. The wound discharged synovia and its borders were covered by a synovial clot. The hair in the neighborhood was cut off, the wound cleaned and the leg placed in a solution of 2°/₀ creolin for the day. In the evening a dressing of iodoform and tannine was applied, under a bandage.

The wound grew worse for eight days; the borders became pale and soft and bled upon the least disturbance. The flowing synovia continued. The ninth day the horse refused his food, for the first time, and would not put the foot to the ground. The borders of the wound were swollen, discolored and hot. There was decided fever; temperature 106, pulse 20. The wound was now treated with strong solutions of corrosive sublimate, injections of a 5°/₀ solution of creolin, subsequent tamponning with iodoform and bandaging aseptically.

The horse was given antifebrine (ʒv per day) and subcutaneous injections of spirits of camphor and caffeine. Two days later it was found down, unable to rise; no appetite; bandage changed, borders of wound not quite so much swollen. The internal treatment was continued. Improvement now commenced, which under the same treatment, led to complete recovery in about a month. *Zeitschrift für Veterinärkunde.* No. 2. iii. 1891.

---

### Cure of a Fistula of the Spermatic Cord by Injections of Iodine.

Army Veterinarian Christ opened in June of last year, an abscess on the scrotum of a young gelding that had been recently purchased. In spite of the treatment the wound would not heal and continued to discharge until February. A firm tumor had, in the meantime, developed on spermatic cord and had reached the size of a child's head. Injections of Lugal's solution were made in eight different portions of this tumor, but the growth did not seem to be affected by the treatment. Hence, a few days later, the horse was cast for the purpose of extirpating tha tumor, when the operator found, much to his own surprise that sloughing had already commenced and the wound had become smaller. The operation was put off and the horse recovered in a short time, without further treatment. *Zeitscrift für Veterinärkunde.* No. 4. iii.

---

The importation of American cattle into Germany is allowed under the following conditions: They must be unloaded at Hamburg and transported at once, in wagons, to Altona, a distance of about 5 miles. Immediately upon their arrival, they have to be slaughtered, before the eyes of the police (to see that none are taken away) and are then inspected by Veterinarians. Other cattle, which come in contact with those from America are not allowed to leave the spot but are slaughtered at once. That is, our cattle are all looked upon as having been exposed to contagious diseases and are subjected to the laws pertaining to these diseases.

---

Cesare recommends a 40 to 50°/o solution of antipyrine, as an hæmostatic. The lung causes a more or less prompt cessation of hæmorrhage from both the large and small vessels by making the blood thicker without coagulating it, and thus hindering its flow from the vessels. *Zeitschrift für Veterinärkunde.* No. iii.

---

An ox was recently slaughtered in Frankfort-on-the-Main, whose kidneys were not well mated. The official slaughterhouse scales showed that one weighed 3, and the other 177 pounds.

---

In the *Chirica Vet.* the following prescription is recommended for constipation of cattle :

℞ Potassii Bitartrat, ℥ vi
Potass. nitr., ℥ ii ss
Gambogæ ʒ iii.

♏. Directions : Divide into 12 parts and give one each three hours in a wine bottle of linseed gruel. *Berl. Thier. Wochs.* No. 29. 1891.

